# Developing Semiautomated Methods to Measure Pre- and Postoperative Syrinx Volumes

**DOI:** 10.3390/jcm12216725

**Published:** 2023-10-24

**Authors:** Eric A. Kohut, Shantelle A. Graff, Samuel H. Wakelin, Martin Arhin, Govind Nair, John D. Heiss

**Affiliations:** 1 Surgical Neurology Branch, National Institute of Neurological Disorders and Stroke, The National Institutes of Health, Bethesda, MD 20892, USA; eak268@cornell.edu (E.A.K.); shw43@georgetown.edu (S.H.W.); martin_arhin@med.unc.edu (M.A.); heissj@ninds.nih.gov (J.D.H.); 2 qMRI Core Facility, National Institute of Neurological Disorders and Stroke, Bethesda, MD 20892, USA; govind.bhagavatheeshwaran@nih.gov

**Keywords:** syringomyelia, Chiari malformation Type 1, spinal cord, surgical outcomes, quantitative magnetic resonance imaging

## Abstract

Neurosurgeons evaluate MRI scans to document whether surgical treatment has reduced syrinx size. Manual measurement of syrinx volume is time-consuming and potentially introduces operator error and bias. Developing convenient semiautomated volumetric analysis methods may encourage their clinical implementation and improve syringomyelia monitoring. We analyzed 30 SPGR axial MRI scans from 15 pre- and postoperative Chiari I and syringomyelia patients using two semiautomated (SCAT and 3DQI) methods and a manual Cavalieri (CAV) method. Patients’ spinal cord and syrinx volumes pre- and postoperatively were compared by paired *t*-test. A decrease in syrinx volume (mm^3^) after surgery was detected across all methods. Mean syrinx volume (± SD) measured by CAV (*n* = 30) was, preoperatively, 4515 mm^3^ ± 3720, postoperatively 1109 ± 1469; (*p* = 0.0004). SCAT was, pre, 4584 ± 3826, post, 1064 ± 1465; (*p* = 0.0007) and 3DQI was, pre, 4027 ± 3805, post, 819 ± 1242; (*p* = 0.001). 3DQI and CAV detected similar mean spinal cord volumes before (*p* = 0.53) and after surgery (*p* = 0.23), but SCAT volumes differed significantly (*p* = 0.005, *p* = 0.0001). The SCAT and 3DQI semiautomated methods recorded surgically related syrinx volume changes efficiently and with enough accuracy for clinical decision-making and research studies.

## 1. Introduction

An intramedullary fluid-filled cyst, or syrinx, characterizes syringomyelia. Syringomyelia is most associated with Chiari I malformation [1]. However, it may also accompany other conditions obstructing cerebrospinal fluid (CSF) flow, including degenerative spinal disorders, arachnoiditis, and other craniovertebral junction abnormalities like basilar invagination [2]. Patients with Chiari I malformation and syringomyelia suffer neurological symptoms like headaches, poor balance, upper extremity weakness, numbness, loss of sensation, and pain. Symptoms may progress and include lower extremity spasticity if the syringes go untreated. Symptoms prompt patients to seek medical treatment. Chiari I malformation and syringomyelia are definitively diagnosed by MRI scanning of the brain and spine.

Typically, a few mL of CSF is expelled from the intracranial cavity into the cervical subarachnoid space in response to brain expansion during cardiac systole. However, in Chiari I malformation-associated syringomyelia, the cerebellar tonsils block normal CSF efflux pathways into the cervical subarachnoid space. This results in the cerebellar tonsils descending caudally in place of CSF during systole. The caudal motion of the cerebellar tonsils acting on a partially enclosed spinal subarachnoid space results in elevated cervical subarachnoid pulse pressure waves. The enlarged cervical subarachnoid pressure waves drive CSF through the spinal cord’s perivascular (Virchow–Robin) spaces. Excess intramedullary fluid coalesces into a syrinx. Enlarged subarachnoid pressure waves on the spinal cord surface propel the syrinx fluid, expanding and lengthening the syrinx [3].

The treatment for symptomatic patients with Chiari I malformation and syringomyelia is posterior fossa decompression surgery, which aims to open the cerebrospinal fluid pathways at the foramen magnum, reduce syrinx size, and arrest the progression of syringomyelia [4,5,6]. Postoperatively, the effectiveness of the decompression surgery to open the CSF pathways and resolve syringomyelia is shown on MRI by a significant reduction, probably more than 50%, in syrinx diameter. Patients whose syringes do not deflate significantly after surgery and have persistent or worsening symptoms are evaluated to decide if the decompression surgery was unsuccessful in enlarging the CSF pathways at the foramen magnum. Reoperation may be necessary if decompression surgery is unsuccessful and offers a good chance of resolving syringomyelia [7].

Enlargement of syrinx diameter increases compression of neural structures, tiny veins, and capillaries within the spinal cord around the syrinx. Developing precise, unbiased, and reproducible tools to measure syrinx size will aid in clinical and research assessment of the amount of change in syrinx size after decompression surgery. The current standard for measuring syrinx volume is the Cavalieri method (CAV), which requires manual measurement of the cross-sectional areas of patients’ syringes on axial MRI slices. The CAV approach is a widely accepted method for measuring the volume of neuroanatomical structures [8,9,10]. CAV is reliable for measuring the volume of anatomical structures before and after treatment but is an arduous method requiring manual drawing of areas, taking 4 or more hours per MRI scan. Manually measuring cross-sectional areas also introduces operator error and possible bias. Developing convenient semiautomated analysis methods may encourage their clinical implementation and improve syringomyelia monitoring. To this end, we compared two semiautomated measurement techniques to the CAV method to see if the semiautomated techniques would be more efficient, less operator-dependent, and sufficiently reliable to aid clinical decision-making and research reliability.

We repurposed software programs and imaging tools for measuring structures in other spinal cord disorders. The Spinal Cord Analysis Tool (SCAT) is an edge detection software developed by the NINDS MRI Facility. Initially used for multiple sclerosis patients, SCAT accurately measures spinal cord atrophy by detecting the differences in pixelated densities within MRI scans—as depicted through alternating contrasts [11]. The three-dimensional quantitative imaging technology developed by Massachusetts General Hospital, 3DQI, effectively analyzes the volumes of neurofibromas and related tumors through MRI pixel densities [12,13]. In patients with syringomyelia associated with Chiari I malformation, we compared the SCAT and 3DQI programs’ measurement of syrinx and spinal cord volumes before and after surgical treatment and compared these methods with the standard Cavalieri volume measurement method (CAV).

## 2. Materials and Methods

### 2.1. Recruitment and Study Design

This data for this clinical study were obtained from research participants in NIH protocol 10N0143, Natural History Study of Patients with Syringomyelia. The NIH Combined Neuroscience Institutional Review Board approved the research protocol: 10N0143. Consent was obtained from all patients. All patients were evaluated and treated at a single clinical site. Eligible participants for this study were required to (1) have syringomyelia or a condition predisposing them to syringomyelia formation, such as Chiari I malformation, (2) be 18 years of age or older, and (3) be able to give informed consent. For patients to be eligible for surgery, they were required to (1) have new or increased impairment in sensation, strength, or walking within the previous 2 years, (2) have an MRI scan showing a syrinx corresponding to a part of the spinal cord that could produce their symptoms, and (3) be in sufficient health to withstand a major surgical procedure and remain active during the recovery period. Participants were excluded from the study if they were pregnant, could not have an MRI scan as determined by the radiologist, or had a bleeding problem that could not be corrected.

A group of 15 patients with Chiari I malformation and syringomyelia received preoperative MRI scans of the cervical, thoracic, and lumbar spine. Postoperatively, following posterior fossa decompression, the same group had MRI scans of the cervical, thoracic, and lumbar spine at varying intervals following posterior fossa decompression. Spine MRIs were performed on Philips Medical Systems Achieva or GE Genesis Signa scanners at 1.5-tesla magnetic field strength. T1 and T2 weighted cervical, thoracic, and lumbar spine scans were recorded from 2010 to 2020. The Picture Archiving and Communication System (PACS) saved and supplied access to all MRI scans. The time range between pre- and post-operation scans was 10–48 months, with a mean gap of 17.7 ± 9.7 months. Cervical, thoracic, and lumbar axial images were aligned on the PACS to ensure continuous cross-sectional measurements.

### 2.2. Manual Cavalieri Method (CAV) of Analysis

**Figure 1:**
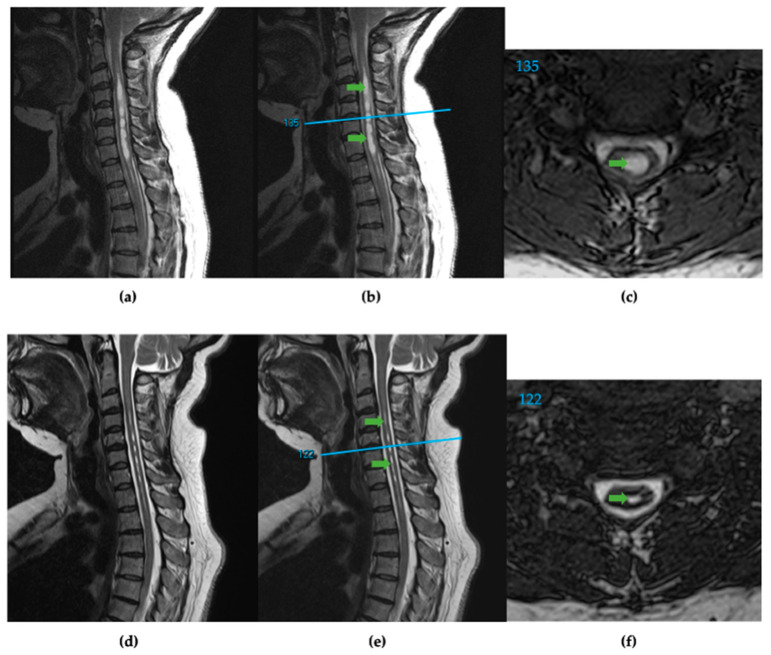
(**a**) T2 weighted sagittal spine scan of subject 91 before surgical treatment. (**b**) Preoperative sagittal spine scan with cervical syringes labeled, green arrows, and the reference line, blue, for axial slice location. (**c**) Preoperative axial slice 135 with labeled syrinx, green. (**d**) T2 weighted sagittal spine scan after surgical treatment. (**e**) Postoperative sagittal spine scan with diminished cervical syringes labeled, green arrows, and the reference line, blue. (**f**) Postoperative axial slice 122 with labeled syrinx cross-section, green.

Magnetic resonance images were stored and retrieved on the PACS. A sagittal cut localizer linked to the axial images was used to find the distance of each axial slice from the foramen magnum (in mm) (Figure 1). The slice thickness of contiguous axial slices ranged from 1–3 mm. Of the 15 preoperative scans analyzed, the slice thickness in 9 patients was 1 mm, in 4 was 2 mm, and in 2 was 3 mm. For the 15 postoperative scans, slice thickness in 7 patients was 1 mm, and in 8 was 2 mm. In the 2 of 15 patients where the pre- and postoperative axial slices’ millimeter increments differed, the common multiple for both increments was used to control the internal precision of documented volumes. The corresponding image and slice numbers were documented. Measured slices started at the foramen magnum and ended at the caudal end of syringes. The Cavalieri method (CAV) used the Freehand Region of Interest (ROI) tool to measure all slices’ spinal cord and syrinx cross-sectional areas. Measurements were then transferred to data spreadsheets with the corresponding image type, slice number, and millimeters from the foramen magnum location. The cross-sectional areas, with corresponding position data points, were graphed to ensure proper spinal cord and syrinx alignment.

### 2.3. Spinal Cord Analysis Tool (SCAT) Method of Analysis

**Figure 2:**
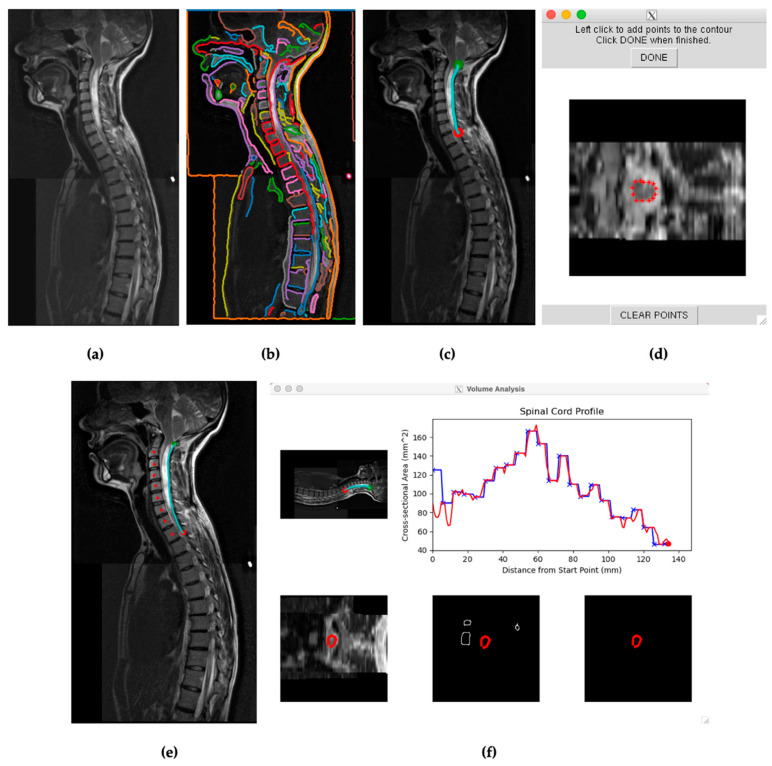
(**a**) Visual from SCAT of a stitched sagittal spine scan for subject 29. (**b**) SCAT program edge detection software presenting continuous edges throughout the T2 scan. Each identified edge is visually represented in a distinct color. (**c**) Selected edge on the posterior surface of the spinal cord encompassing the length of the syrinx in the cervical spine between the green and red points. (**d**) Initial calibration for SCAT to measure edges across axial slices. (**e**) Mapping the spine for vertebral analysis. (**f**) Initial cross-sectional measurements of subject 29′s spinal cord across the selected edge before, blue, and after, red, vertebral analysis.

The same patient data scans and corresponding stitched DICOM information were uploaded to protected servers and accessed through a BASH shell. Through the shell, modified Spinal Cord Analysis Tool (SCAT) scripts, previously used to characterize changes in spinal cord volumes for multiple sclerosis patients, were accessed [11]. The SCAT program was launched in an environment containing the uploaded scans. SCAT initially presented stitched sagittal scans of each patient’s spinal cord. The program then presented the detectable edges based on image contrasts and pixel densities. The contrasts were adjusted to detect patients’ spinal cords’ most extended continuous edges (Figure 2b).

Within the presented sagittal scans, a continuous edge, passing across the anterior or posterior edge of the spinal cord, was selected in all scenarios. Each measurement began at the foramen magnum. Data points were then selected across these edges, caudal to the most caudal syrinx. The approach for measuring spinal cords was like that of previously studied multiple sclerosis patients [8]. When selecting an edge, smaller continuous edges within the spinal cord with detected contrasts within the syrinx were avoided, allowing SCAT to differentiate spinal cord edges from the syrinx. The axial slices were then semiautomatically measured across the spinal cord edges. As the program collected data points, the operator adjustments ensured that the edge was calibrated to the spinal cord, not the syrinx.

After receiving visual representations of the first pass data collections, the operator reviewed the calibrated edges to ensure the spinal cord was detected across axial values. Individual data points that incorrectly detected spinal cord or syrinx edges on axial slices were then manually recalibrated and reincorporated into the data results. The vertebral analysis option was selected for SCAT analyses extending over multiple vertebrae and spinal cord curvatures. The vertebral analysis option was selected when values did not visually differ significantly from linear trends on the first pass data collections. Measurements were then saved to the server and later transferred to Excel files. Spinal cord measurements began at the foramen magnum, as initially chosen by the green marker within the program, and the measured edges ended at the red marker (Figure 2c).

Patients’ syringes were measured by SCAT, accessing the same scans used for spinal cord detection and measurement. Image contrast was increased to highlight the syrinx and allow SCAT to detect its edges better. SCAT then analyzed adjusted sagittal scans for continuous edges. Edges were then selected that either (1) outlined a more extensive continuous syrinx or (2) detected an edge in either the anterior or posterior side of the spinal cord that outlined a continuous syrinx. The edge extending from the cranial segment (green marker) to the most caudal segment (red marker) ran along the longest detectable continuous syrinx, Figure 2c. The SCAT program was started after the first slices were calibrated manually to detect the syrinx, prompting the program to continue measuring the syrinx with the selected edge. This procedure was performed for both preoperative and postoperative scans. Some postoperative syringes decreased considerably or disappeared compared to preoperatively. Even with adjusted contrast, the SCAT program could not detect a syrinx in these cases due to insufficient syrinx diameter or continuous edge. These syringes that were nondetectable by SCAT were considered to have zero (0.0) cross-sectional areas and volumes.

### 2.4. Three-Dimensional Quantitative Imaging Technology (3DQI) Method of Analysis

**Figure 3:**
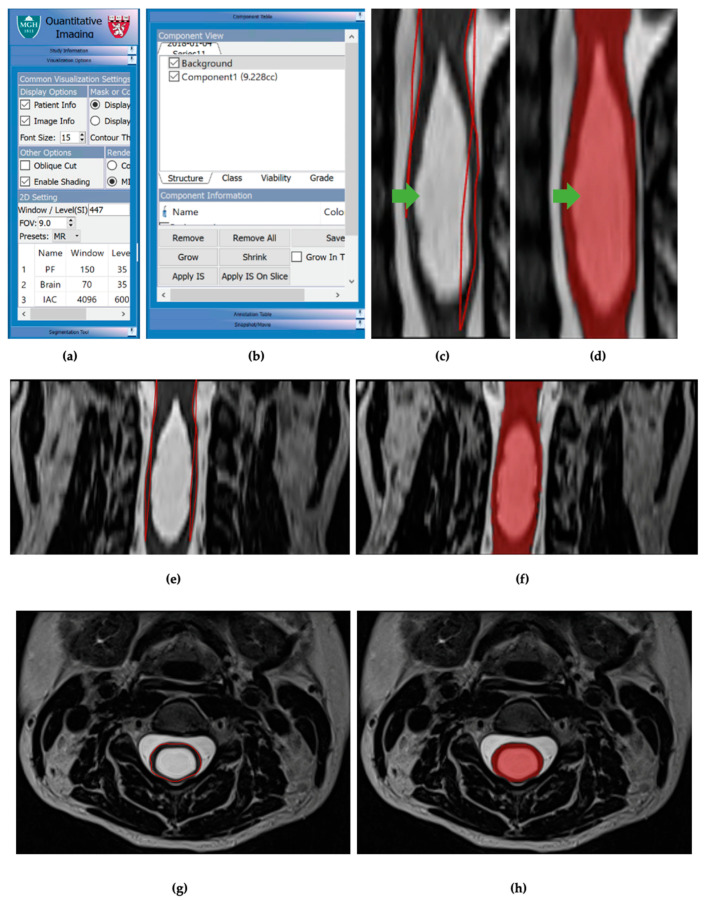
(**a**) Visualization options table for 3DQI imaging. (**b**) Component table for 3DQI measurements. (**c**) Initial 3DQI outline, in red lines, for sagittal slice of subject 161 spinal cord with syrinx, labeled with green arrow. (**d**) 3DQI area measurement, in red highlight, for subject 161 spinal cord with syrinx, labeled with green arrow. (**e**) Expanded view of 3DQI outline, in red lines, for cervical spinal cord on subject 161 pre-operation, sagittal slice. (**f**) Expanded view of 3DQI area measurement for cervical spinal cord on subject 161 pre-operation, sagittal slice. (**g**) 3DQI outline, red line, for subject 161 spinal cord pre-operation, axial slice. (**h**) 3DQI area measurement, in red highlight, for subject 161 spinal cord pre-operation, axial slice.

Image analysis was then performed for the same 15 patients, with controlled scan dates, using the volumetric image analysis program 3DQI (https://3dqi-lab.github.io/3dqi_website/ (accessed on 20 October 2023)). The 3DQI program has been used to segment tumors and calculate tumor image texture features. All measurements were done on anonymized MRIs. Identical syringes and MRI slices (1–3 mm thickness) were used for volumetric analysis (Figure 3).

### 2.5. Data Management

All images were acquired using a standard protocol and saved in DICOM format on PACS. The images were then loaded into 3DQI software for volumetric analysis. The software segmented each MRI slice’s region of interest (ROI). The cross-sectional area of each MRI slice was also measured using the software. The segmentation process involved using a combination of manual and semiautomated techniques. The ROI (syrinx or spinal cord) was selected on multiple MRI slices in the program. Once selected, the software automatically calculated each slice’s cross-sectional area and volume.

The data from the three methods for spinal cord and syrinx cross-sectional areas relative to the foramen magnum were then aligned for analysis in adjacent columns in Excel. The foramen magnum starting point, line number, slice length (1–3 mm), and syrinx position on PACS images allowed the operators to align the data accurately. The data points were then graphed relative to the same positions and slice distances. The difference in ascertainment of the level of the foramen magnum across methods was corrected to align the data sets [14].

**Figure 4:**
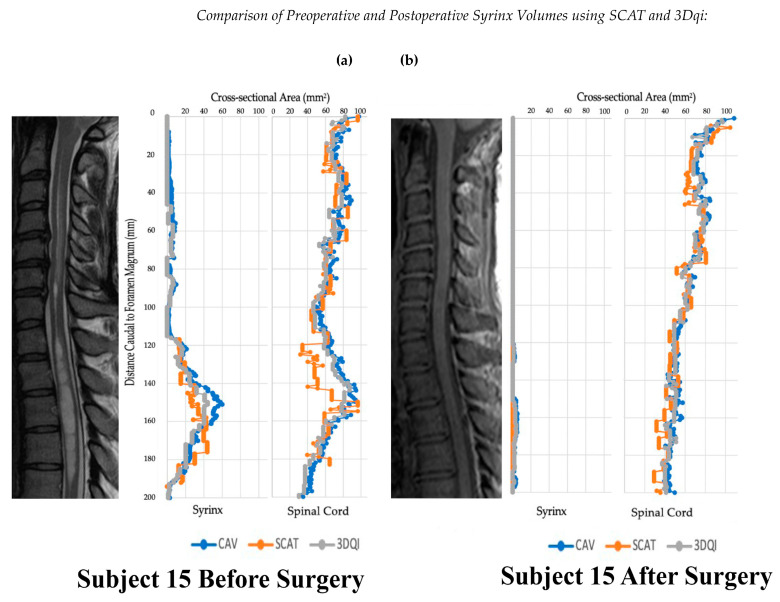
Graphs of subject 15 before and after operation alongside T2 (**a**) and T1 (**b**) weighted sagittal scans of the cervical spine.

The cross-sectional areas for all slices were then summated and multiplied by the image slice thickness (1–3 mm) to calculate syrinx and spinal cord volumes [15,16]. The pre- and postoperative patient volumes using each syrinx volume method were compared using the paired *t*-test. Mean volumes for each circumstance were matched and compared for the same patients. The mean and standard deviation for syrinx and spinal cord volumes for the pre- and postoperative syrinx groups were calculated. The two semiautomated measurement methods, SCAT and 3DQI, were compared to CAV using the paired *t*-test. Data were also graphically analyzed to visualize dimensional changes in the spinal cord and syrinx that resulted from surgical treatment (Figure 4).

### 2.6. Formatting of Mathematical Components

Variables and equation for summating cross-sectional areas to find syrinx and spinal cord volumes:

Variables:

a = volume in mm^3^
b, c, d, e, f, … = axial slice cross-sectional areas
x = thickness in mm of axial slices

Equation for Summation:

a = b(x) + c(x) + d(x) + e(x) + f(x) + … = (x)(b + c + d + e + f + ….)

### 2.7. Statistical Analyses

Paired quantitative volume variables were compared using a paired-sample *t*-test and GraphPad Prism Version 9.4.1(458) software. Interoperator and intraoperator consistency was evaluated by interclass correlation analysis using the RStudio Version 4.3.1 program.

#### Interoperator and Intraoperator Consistency

We completed repeated measurements of three individual cases of various syrinx volumes to assess the inter- and intraoperator consistency of SCAT and 3DQI measurements. Cases 9 (small syrinx), 161 (moderate syrinx), and 113 (large syrinx) were measured by operators E.K., M.A., and S.G. Consistency between these independent operators was measured using an interclass correlation coefficient analysis (ICC). The same operator assessed three cases of different syrinx volumes (73, small, 84, moderate, and 156, large) on different dates to evaluate intraoperator reproducibility. Consistency within operators was also measured using ICC analysis. The ICC quantifies the degree of similarity or agreement between multiple measures; its values range from 0 to 1. An ICC of 0 indicates no agreement between measures, while 1.0 indicates perfect agreement. ICC values and their corresponding 95% confidence interval (upper and lower limits) were reported. The upper and lower limits of the confidence interval create a range of values that estimate where the true ICC value falls. Variability was then quantified by calculating the percent difference between and within operators.

## 3. Results

### 3.1. Intra- and Interoperator Variability

The overall intraoperator ICC was 0.98 [95% confidence interval: 0.94, 0.99]. SCAT had the highest variability between measurement sessions conducted by the same operator (14%). The overall interoperator ICC was 0.99 [95% confidence interval: 0.96, 1.0]. SCAT showed the highest variability among different operators (14%). CAV and 3DQI had low interoperator variability at 5% and 3%, respectively. Both methods also resulted in low intraoperator variability (CAV: 0.14%, 3DQI: 1%).

### 3.2. Accuracy of Volumetric Analysis Using SCAT and 3DQI

CAV, SCAT, and 3DQI were used to measure each patient’s preoperative and postoperative syrinx and spinal cord volumes. For spinal cord volumes, all methods detected a statistically significant difference between preoperative and postoperative spinal cord volumes. Specific findings were CAV (CAV preoperative: 13,399 mm^3^ ± 6212, CAV postoperative: 8722 ± 3389, *p* = 0.0019), SCAT (SCAT preoperative: 11,325 mm^3^ ± 5729, SCAT postoperative: 7530 ± 3209, *p* = 0.0035), and 3DQI (3DQI preoperative: 12,935 mm^3^ ± 6213, 3DQI postoperative: 8280 mm^3^ ± 3315). For syrinx volumes, all methods detected a statistically significant difference between preoperative and postoperative syrinx volumes. Syrinx volume values were CAV (CAV preoperative: 4515 mm^3^ ± 3720, CAV postoperative: 1109 mm^3^ ± 1469, *p* = 0.0004), SCAT (SCAT preoperative: 4584 mm^3^ ± 3826, SCAT postoperative: 1064 mm^3^ ± 1465, *p* = 0.0007) and 3DQI (3DQI preoperative: 4027 mm^3^ ± 3805, 3DQI postoperative: 819 mm^3^ ± 1242, *p* = 0.0010) (Table 1).

The SCAT and 3DQI methods were compared to the CAV method to decide if the syrinx and spinal cord volumes recorded for the semiautomated SCAT and 3DQI methods differed significantly from the manual (CAV) method. Preoperative and postoperative syrinx and spinal cord volumes for each patient were measured (Table 2). Compared to CAV, SCAT produced lower values for the volume of the spinal cord on both preoperative (CAV, 13,399 mm^3^ ± 6212; SCAT, 11,325 ± 5729, (*p* = 0.004)); and postoperative (CAV, 8722 ± 3389; SCAT, 7530 ± 3209; *p* = 0.0001) MRI scans. However, SCAT values for syrinx volume were like CAV; preoperative (CAV: 4515 mm^3^ ± 3720, SCAT 4583 mm^3^ ± 3826; *p* = 0.89) and postoperative (CAV: 1109 mm^3^ ± 1469, SCAT: 1064 mm^3^ ± 1465; 0.82) (Table 1). Compared to CAV, 3DQI recorded similar spinal cord volumes for both preoperative (CAV: 13,399 mm^3^ ± 6212, 3DQI: 12,935 mm^3^ ± 62,123; *p* = 0.53) and postoperative (CAV: 8722 ± 3389, 3DQI: 8279 mm^3^ ± 3315; 0.23) MRI scans. 3DQI and CAV had similar values for the volume of preoperative syringes (CAV: 4515 mm^3^ ± 3720, 3DQI: 4026 mm^3^ ± 3805). However, after surgery, when syringes were smaller, 3DQI reported lower volumes than CAV for postoperative syringes (CAV: 1109 mm^3^ ± 1469, 3DQI: 819 mm^3^ ± 1242; 0.0019) (Table 1). Both semiautomated methods showed changes in syrinx and spinal cord volume after successful craniocervical decompression surgery for patients with Chiari I malformation and syringomyelia.

## 4. Discussion

### 4.1. Limitations

Semiautomated volumetric tools have the potential to revolutionize the field of medical imaging by providing an efficient and accurate means of measuring tumors and other anatomical volumes. However, they are not without their limitations. One limitation is user-dependent variability. Variations in image resolution, noise, and artifacts can significantly impact the accuracy of tumor and other volume measurements. When anatomical borders are ill-defined, obscured by surrounding structures, or disrupted by imaging artifacts, semiautomated tools and independent operators may struggle to provide precise measurements. Thus, these tools often require human intervention for tasks such as delineating anatomical boundaries or refining the contours generated by the software. Since users may interpret images differently, this can result in variations in tumor and other volume calculations. Recognizing this limitation is most important when using SCAT for analysis, which resulted in the highest interoperator variability of 14%, followed by CAV’s 5% and 3DQI’s 3%. Additionally, this highlights the importance of using high-quality imaging techniques when using these semiautomated analysis methods, especially when using SCAT. As depicted in Figure 2d, SCAT greatly compresses image files, making it challenging for an independent operator to delineate organic boundaries. Reasonable access to quality imaging resources should be considered when assessing the feasibility of using these semiautomated methods.

Another limitation is the level of expertise needed to effectively utilize semiautomatic volumetric tools like 3D quantitative imaging (3DQI) and SCAT. A basic understanding of the software interface and medical imaging principles may suffice in simple cases featuring well-defined tumors or syringes and high-quality imaging. Radiologists, oncologists, or those with rudimentary knowledge in these areas can often navigate the software to generate accurate syrinx volume measurements. As the complexity of a radiological image increases, so does the level of expertise required. Consequently, proficiency in image interpretation and anatomy is essential to identify syrinx boundaries and manually adjust the automated contours accurately.

### 4.2. Discussion

Relief of spinal cord distension and decreased postoperative syrinx size are associated with clinical improvement after posterior fossa decompression [17]. Additionally, unchanged or increasing postoperative syrinx volume are signs of ineffective decompression surgery. Reoperation is needed in patients with worsening symptoms and persistent syringomyelia [18]. The Cavalieri method for measuring spinal cord syringes is exact but impractical for clinical use because it is time-consuming. Although this method is the gold standard for analyzing syrinx volume in syringomyelia, semiautomated approaches may give clinicians and researchers a more rapid way to calculate syrinx volume.

SCAT was initially developed by investigators in the NINDS Viral Immunology Section and Quantitative MRI Core Facility for determining spinal cord cross-sectional area in neuroinflammatory diseases [11,19]. The SCAT method in our study was adapted to determine the cross-sectional area of the syrinx and spinal cord pre- and postoperatively. This method is better than manual methods for cross-sectional area measurement because it finds the cross-sectional area perpendicular to the cord at each point. The CAV method, in contrast, will record larger cross-sectional areas than SCAT in areas where spinal curvature results in axial images cutting obliquely rather than perpendicularly through the spinal cord or syrinx. The SCAT method also preprocesses and resamples the images to 1 mm isotropic resolution, whereas CAV does not use image preprocessing. 

The SCAT method accurately measured preoperative and postoperative volumes of moderate-sized syringes, showing this program’s potential usefulness in analyzing treatment-related changes in syrinx volume. SCAT analyzed syrinx volumes significantly faster than the CAV method. The SCAT method was like CAV in detecting differences in the preoperative and postoperative syrinx volumes.

The SCAT method could not detect or accurately measure small postoperative syringes. SCAT had limited ability to measure small syrinx CSA because the program needs a specified number of points to detect a structure like a syrinx. Improving image resolution improves SCAT measurement accuracy. However, in cases in which surgical treatment results in such a dramatic reduction in syrinx volume that SCAT cannot detect the syrinx after surgery, there is little question about the effectiveness of treatment. SCAT was less exact than CAV in measuring very large spinal syringes. There were also significant differences in CAV and SCAT measurements of preoperative and postoperative spinal cord volumes (Table 1). For syrinx studies, SCAT is acceptably exact in measuring moderate-sized syringes, but CAV is more exact than SCAT in large and small syringes.

SCAT is an edge detection program and could not continuously analyze multiple syringes because the edge of the reference syrinx did not extend across the gaps to other cord syringes. SCAT measurement of a patient’s most extensive continuous syrinx resulted in volumes like the CAV measurement of the largest continuous syrinx. However, SCAT did not record the syrinx volume contained in smaller syrinx components that were discontinuous with the largest syrinx component. Although it is possible to execute SCAT multiple times to measure each syrinx in a patient’s spinal cord individually, doing so would be time-consuming like CAV, defeating the purpose of using a semiautomated method. SCAT has features reducing its clinical acceptance. The SCAT program requires high-quality imaging techniques and an operator’s basic coding knowledge. Additionally, the SCAT program had more intraoperator and interoperator variability than the 3DQI and CAV methods because the operators saw the syrinx edge less clearly. Despite these limitations, SCAT detected significant differences in pre- and postoperative syringes.

The 3DQI program produced similar values to CAV, except that CAV reported larger volumes for small postoperative syringes. The ability of 3DQI to accurately measure and detect a significant difference in preoperative and postoperative spinal cord volumes may supply a clinical benefit since decreased spinal cord distension is a crucial factor in patients showing postoperative improvement [17]. Although indirect, the difference in preoperative and postoperative spinal cord volumes represents the syrinx fluid drained from spinal cord syringes postoperatively. The 3DQI program detected the difference in preoperative and postoperative syrinx volumes and accurately measured spinal cord volumes. Clinicians interested in quickly and accurately measuring spinal cord and syrinx volumes may wish to use this program, as it is acceptably accurate and efficient.

Measurements of inter- and intrarater reliability were conducted in this study. Notably, all three programs demonstrated exceptional consistency in their measurements. We found that SCAT measurements were the most variable, both within and between operators, while 3DQI’s were less variable. Before applying these semiautomated programs clinically, future investigators would be advised to examine their reliability across operators of varying radiographic expertise and institutions. In our study, all measurements were made by investigators from the same institution. Radiologists, neuroradiologists, neurologists, neurosurgeons, and imaging scientists have the expertise to use these programs and reliably measure the volume of anatomical structures. In a recent International Consensus Document on the diagnosis and treatment of Chiari malformation and syringomyelia in adults, the Vaquero Index was reported among the evidence-based statements, with a 96.3% agreement [20]. The semiautomated methods of measuring syrinx and spinal cord volumes presented in this study could be compared with other radiographic measurements (i.e., the Vaquero Index) [21,22] and clinical outcomes in future international collaborative studies among centers with expertise treating patients with Chiari I malformation and syringomyelia.

## 5. Conclusions

The SCAT and 3DQI programs measured syrinx and spinal cord volume significantly faster than the Cavalieri method. Furthermore, they supplied acceptably exact data and workable methods for assessing the reduction in syrinx volume as a surgical outcome. CAV and 3DQI were better than SCAT in including the syrinx volume component contained in irregular, discontinuous, and sub 2 mm diameter syringes. While both semiautomated methods were sufficiently accurate, SCAT was more variable and less user-friendly than 3DQI. 3DQI may be a more practical tool for monitoring syringomyelia within clinical settings. Furthermore, its ability to measure changes in spinal cord volume may make this technology applicable to diagnosing and monitoring spinal cord atrophy after treatment of syringomyelia [23].

## Figures and Tables

**Table 1:** Comparison of the mean spine and syrinx volumes for aligned spinal cord segments before and after operation, measured by each method. *p*-values for *t*-tests of the semiautomated programs compared to the manual Cavalieri method standard.

Volumes (mm^3^)								
	CAV Mean	CAV SD	SCAT Mean	SCAT SD	*p*-Value CAV vs. SCAT	3DQI Mean	3DQI SD	*p*-Value CAV vs. 3DQI
Pre-op Syrinx Volume	4515	3720	4584	3825	0.891	4027	3805	0.117
Post-op Syrinx Volume	1109	1469	1064	1465	0.8200	819	1242	0.0019 *
Pre-Minus Post Syrinx Volume (*p*-value)	3406 (0.0039 †)		3520 (0.0065 †)			3208 (0.00096 †)		
Pre-op Spinal Cord Volume	13,399	6212	11,325	5729	0.0051 *	12,935	6213	0.533
Post-op Spinal Cord Volume	8722	3389	7340	3210	0.0001 *	8279	3315	0.228
Pre-Minus Post Spinal Cord Volume (*p*-value)	4677 (0.0071 §)		3985 (0.0035 §)			4656 (0.0071 §)		

* Significant difference paired *t*-test. CAV vs. 3DQI method. † Significant difference, paired *t*-test: pre- vs. post-surgical syrinx volume. § Significant difference, paired *t*-test: pre- vs. post-surgical spinal cord volume.

**Table 2:** The mean volumes collected from aligned spinal cord measurements across methods, before and after surgical treatment, and the mean volumes of detectable syringes.

Means	Subject Number	Volume (mm^3^) CAV	Volume (mm^3^) SCAT	Volume (mm^3^) 3DQI
Aligned Pre-operation Spinal Cord	9	2107	1933	2415.2
	15	12,982.7	11,599.7	11,919.1
	27	22,116.5	15,658.9	12,140.84
	29	14,996.9	13,892.5	14,957
	73	5054.8	3837	5290.5
	84	13,050.5	10126	14,502.9
	91	6987.1	6596	5941.12
	101	9818.1	7692.6	9508.2
	113	17,363.7	14,557.8	16,301.29
	139	19,171.4	21,825.8	19,743
	156	22,506.4	20,845.8	24,217
	161	9573	8888.5	9082.58
	168	20,192.7	15,604.9	21,337.5
	169	15,369.3	8587.5	17,310.9
	177	9697	8228.25	9350.7
Aligned Post-operation Spinal Cord	9	2008.1	1945.8	2094.8
	15	15,534.4	14,333.9	15,720.6
	27	9948.7	8476	9971.2
	29	7282.3	6994.9	7324.7
	73	3696.6	2703.3	3826.1
	84	8656	6420.1	9405.1
	91	5894.2	4783.5	5228.4
	101	10,623.6	8054	9881.7
	113	11,246	8740	10,088.1
	139	12,554	12,530.5	11,085.24
	156	10,107.2	9272.8	11,330
	161	6489	5768.8	7093.8
	168	9569.6	7059.8	9639.4
	169	8329	7760.5	9061
	177	8885.8	8109	8404.9
Total Pre-operation Syrinx	9	261.7	459	0
	15	2269	1969.6	1850.3
	27	7806.6	8500.4	6919
	29	3901.2	6224	3623
	73	746.2	842.6	797.9
	84	5811.5	5538.9	5463.3
	91	770	1632.5	710
	101	661	503.5	570.2
	113	2729	2430.3	2431.45
	139	7827.4	12,166	8167.88
	156	13,528.2	11,390	13,853.8
	161	4117	3606.5	3618.5
	168	7305.9	6952.5	7333
	169	4406.7	4732.5	4263.4
	177	1490	1804.5	802.2
Total Post-operation Syrinx	9	0	0	0
	15	325.5	0	192.2
	27	2647	1915.3	2189
	29	1528.2	0	1319
	73	26.4	0	0
	84	116.9	231.75	96.5
	91	260.8	0	122.2
	101	240.2	0	33.81
	113	455	682.3	183.8
	139	947	2553.8	518.6
	156	5583	4852.3	4649.6
	161	1923.4	2135	1347.7
	168	260.8	0	0
	169	745.6	863.3	841.1
	177	1572.6	2732.3	794.9

## Data Availability

The measurement data presented in this study are available on request from the corresponding author. The imaging data are not publicly available to protect research subject confidentiality.

## Additional Notes for Reference

The spinal cord segments holding the syrinx were viewed before and after surgery on MRI scan images on the PACS. This preliminary step provided operators with a quick reference for where to sample the spinal cord to measure the cross-sectional area of the syrinx and spinal cord by semiautomated methods. The spinal cord segments were aligned using vertebral landmarks. For each patient, the semiautomated methods analyzed segments of the spinal cord of approximately the same length before and after surgery.
